# Nonadherence to Any Prescribed Medication Due to Costs Among Adults with HIV Infection — United States, 2016–2017

**DOI:** 10.15585/mmwr.mm6849a1

**Published:** 2019-12-13

**Authors:** Linda Beer, Yunfeng Tie, John Weiser, R. Luke Shouse

**Affiliations:** 1 Division of HIV/AIDS Prevention, National Center for HIV/AIDS, Viral Hepatitis, STD, and TB, CDC.

The United States spends more per capita on prescription drugs than do other high-income countries (*1*). In 2017, patients paid 14% of this cost out of pocket (*2*). Prescription drug cost-saving strategies, including nonadherence to medications due to cost concerns, have been documented among U.S. adults (*3*) and can negatively affect morbidity and, in the case of persons with human immunodeficiency virus (HIV) infection, can increase transmission risk (*4*,*5*). However, population-based data on prescription drug cost-saving strategies among U.S. persons with HIV are lacking. CDC’s Medical Monitoring Project* analyzed cross-sectional, nationally representative, surveillance data on behaviors, medical care, and clinical outcomes among adults with HIV infection. During 2016–2017, 14% of persons with HIV infection used a prescription drug cost-saving strategy for any prescribed medication, and 7% had cost saving–related nonadherence. Nonadherence due to prescription drug costs was associated with reporting an unmet need for medications from the Ryan White AIDS Drug Assistance Program (ADAP), not having Medicaid coverage, and having private insurance. Persons who were nonadherent because of cost concerns were more likely to have visited an emergency department, have been hospitalized, and not be virally suppressed. Reducing barriers to ADAP and Medicaid coverage, in addition to reducing medication costs for persons with private insurance, might help to decrease nonadherence due to cost concerns and, thus contribute to improved viral suppression rates and other health outcomes among persons with HIV infection.

The Medical Monitoring Project uses a two-stage sample design: 1) states and territories and 2) persons with a diagnosis of HIV infection. Response rates were 100% (states and territories) and 40% (persons). Data were collected using face-to-face or telephone interviews and medical record abstraction during June 2016–May 2017. Data were weighted for unequal selection probabilities and nonresponse. Using data from 3,948 persons taking prescription drugs, the prevalence of prescription drug cost-saving strategies among U.S. adults with HIV with accompanying 95% confidence intervals (CIs) was estimated overall and by selected sociodemographic characteristics. Differences in clinical outcomes between those who did and did not have prescription drug cost saving–related nonadherence were also assessed. Prevalence ratios with predicted marginal means were used to evaluate significant (p<0.05) differences between groups. SAS software (version 9.4; SAS Institute) was used to conduct all analyses.

Persons taking prescription drugs were asked about their use of six cost-saving strategies over the past 12 months: 1) asking a doctor for a lower-cost medication, 2) buying prescription drugs from another country, 3) using alternative therapies, 4) skipping doses, 5) taking less medicine, and 6) delaying filling a prescription because of cost. Interviewees were asked about all prescription drugs, not solely antiretroviral medications. Cost saving–related nonadherence was defined as using any of the latter three strategies (*3*). Persons who reported needing but not receiving medications from ADAP were categorized as having an unmet need for ADAP. All examined covariates were self-reported, except viral suppression and care engagement, which were based on medical record abstraction. All were measured over the previous 12 months except where otherwise noted.

Overall, approximately 14% (95% CI = 12–15) of U.S. adults with HIV used any medication cost-saving strategy, including 7% (95% CI = 6–8) who reported cost saving–related nonadherence; among this group, 4% (95% CI = 3–5) skipped doses, 4% (95% CI = 3–5) took less medicine, and 6% (95% CI = 5–7) delayed a prescription. In addition, 9% (95% CI = 7–10) asked a doctor for lower-cost medicine, 1% (95% CI = <1–1) bought drugs from another country, and 2% (95% CI = 2–3) used alternative medicine. Cost saving–related nonadherence was not associated with age, race/ethnicity, gender, homelessness, or time since HIV diagnosis (Table 1). Household income above the poverty level was associated with nonadherence due to prescription drug costs (8% versus 5%). Nonadherence due to prescription drug costs was higher among persons with a disability (9%) than among those with no disability (5%). Among those with health insurance, cost saving–related nonadherence was more likely among persons with private insurance (8%) than among those who did not have private insurance (6%) and was less likely among those with Medicaid (5%) than among those who did not have Medicaid (8%). Persons who had an unmet need for medications from ADAP were approximately five times as likely to be nonadherent because of cost (32%) than were those who received ADAP (7%, prevalence ratio = 5).

**TABLE 1:** Prevalence of nonadherence to any prescribed medication due to costs among persons with human immunodeficiency virus (HIV) infection who were taking prescription medications (N = 3,948), by sociodemographic characteristics — Medical Monitoring Project, 2016–2017

Characteristic*	Cost savings–related nonadherence		Prevalence ratio (95% CI)	P-value
	No.^†^	% (95% CI)^§^		
**Total**	**252**	**7.0 (5.8–8.3)**	**—**	**—**
**Age group (yrs)**				0.91
18–39	57	7.1 (4.9–9.3)	1.1 (0.7–1.7)	
40–49	60	7.6 (4.6–10.5)	1.1 (0.7–2.0)	
≥50	135	6.7 (4.5–8.9)	Reference	
**Race/Ethnicity**				0.05
White, non-Hispanic	97	8.7 (6.9–10.4)	1.5 (0.8–2.6)	
Black, non-Hispanic	97	6.2 (4.8–7.6)	1.0 (0.6–1.9)	
Hispanic or Latino	37	5.9 (2.6–9.3)	Reference	
Other/Multiracial^¶^	21	8.2 (4.9–11.6)	1.4 (0.8–2.6)	
**Gender**				0.44
Male	180	6.9 (5.4–8.4)	Reference	
Female	70	7.8 (5.8–9.7)	1.1 (0.8–1.5)	
**Education**				0.19
<High school	29	4.8 (2.7–6.8)	Reference	
High school diploma or equivalent	52	6.5 (3.9–9.2)	1.4 (0.8–2.4)	
>High school	171	7.9 (6.0–9.9)	1.7 (1.0–2.9)	
Poverty level**				<0.01
Above poverty level	155	8.3 (6.5–10.2)	1.6 (1.2–2.1)	
At or below poverty level	78	5.3 (4.0–6.7)	Reference	
**Homeless**				0.21
Yes	29	9.1 (5.9–12.2)	1.3 (0.9–2.0)	
No	223	6.9 (5.5–8.2)	Reference	
**Years since HIV diagnosis**				0.92
<5	36	6.7 (4.7–8.7)	Reference	
5–9	52	7.4 (4.9–9.8)	1.1 (0.7–1.7)	
≥10	164	7.1 (5.3–8.8)	1.1 (0.7–1.6)	
**Any disability** ^††^				<0.01
Yes	156	9.3 (7.1–11.4)	1.8 (1.4–2.5)	
No	96	5.1 (3.8–6.3)	Reference	
**HIV disease stage 3**				0.63
Yes	154	6.8 (5.1–8.5)	Reference	
No	98	7.4 (5.5–9.3)	1.1 (0.8–1.5)	
**Any private insurance among insured**				0.01
Yes	109	8.3 (6.6–10.1)	1.5 (1.1–2.0)	
No	109	5.7 (4.1–7.2)	Reference	
**Any Medicaid coverage among insured**				<0.01
Yes	89	5.0 (4.0–5.9)	Reference	
No	131	8.4 (6.0–10.8)	1.7 (1.2–2.4)	
**Any Medicare coverage among insured**				0.22
Yes	82	7.8 (5.2–10.4)	Reference	
No	138	6.3 (5.1–7.5)	0.8 (0.6–1.1)	
**Any Ryan White HIV/AIDS Program coverage**				0.70
Yes	122	6.7 (5.3–8.0)	Reference	
No	121	7.0 (5.2–8.9)	1.1 (0.8–1.4)	
**Use of AIDS Drug Assistance Program (ADAP)**				<0.01
Received	123	6.7 (5.4–8.0)	Reference	
Needed but did not receive	29	31.5 (16.2–46.8)^§§^	4.7 (2.8–7.7)	
Did not need or receive	95	5.9 (4.3–7.5)	0.9 (0.7–1.2)	

**Abbreviations:** AIDS = acquired immunodeficiency syndrome; CI = confidence interval.

* All variables assessed during the 12 months before the survey; all data were self-reported.

^†^ Numbers are unweighted.

^§^ Percentages and corresponding CIs are weighted percentages.

^¶^ Includes American Indian/Alaska Native, Asian, Native Hawaiian/Other Pacific Islander, or multiple races.

** Poverty guidelines as defined by the U.S. Department of Health and Human Services.

^††^ Self-reported problems with hearing, vision, cognition, mobility, self-care, or independent living.

^§§^ CI ≥0.30 and should be interpreted with caution.

Persons with cost saving–related nonadherence were also less likely to be virally suppressed at their last viral load test (64%) and at all tests during the past year (55%) than were those without cost saving–related nonadherence (76% and 68%, respectively) (Table 2). Nonadherence due to prescription drug costs was also associated with lower likelihood of HIV care engagement and higher numbers of emergency department visits and hospitalizations.

**TABLE 2:** Prevalence of clinical characteristics* by nonadherence to any prescribed medication due to costs among persons with human immunodeficiency virus (HIV) who were taking prescription medications (N = 3,948) — Medical Monitoring Project, United States, 2016–2017

Characteristic	No cost savings–related nonadherence		Cost savings–related nonadherence		Prevalence ratio (95% CI)	P-value
	No.^†^	% (95% CI)^§^	No.^†^	% (95% CI)^§^		
**Viral suppression at last test**^¶,^**						
Yes	2,929	75.6 (72.9–78.2)	180	64.3 (54.2–74.5)	0.9 (0.7–1.0)	0.02
No	767	24.4 (21.8–27.1)	72	35.7 (25.5–45.8)	1.5 (1.1–2.0)	0.02
**Viral suppression at all tests in past 12 months**^¶,^**						
Yes	2,634	68.2 (65.9–70.5)	152	55.3 (46.4–64.1)	0.8 (0.7–1.0)	<0.01
No	1,062	31.8 (29.5–34.1)	100	44.7 (35.9–53.6)	1.4 (1.1–1.7)	<0.01
HIV care engagement**^,††^						
Yes	3,155	83.1 (80.8–85.4)	198	68.6 (60.5–76.8)	0.8 (0.7–0.9)	<0.01
No	459	16.9 (14.6–19.2)	51	31.4 (23.2–39.5)	1.9 (1.4–2.5)	
**Hospitalizations**						
0	3,053	83.2 (81.4–85.1)	186	74.5 (64.0–85.0)	0.9 (0.8–1.0)	0.04
1	365	9.9 (8.7–11.2)	34	14.8 (6.5–23.1)	1.5 (0.9–2.6)	0.17
≥2	269	6.8 (5.7–8.0)	32	10.7 (6.3–15.1)	1.6 (1.1–2.2)	0.01
**Emergency department visits**						
0	2,327	63.2 (60.9–65.5)	127	50.3 (38.2–62.3)	0.8 (0.6–1.0)	0.03
1	644	17.4 (15.8–19.0)	49	17.4 (12.5–22.4)	1.0 (0.7–1.4)	0.98
≥2	716	19.4 (17.9–21.0)	75	32.3 (22.3–42.2)	1.7 (1.2–2.2)	<0.01

**Abbreviation:** CI = confidence interval.

* All variables assessed during the 12 months before the survey; all data were self-reported, except where otherwise noted.

^†^ Numbers are unweighted.

^§^ Percentages and corresponding CIs are weighted percentages.

^¶^ <200 copies of viral RNA/mL.

** Ascertained by medical record abstraction.

^††^ Receipt of at least two elements of outpatient HIV care (i.e., encounter with an HIV care provider [could also be self-reported], viral load test result, CD4 test result, HIV resistance test or tropism assay, antiretroviral therapy prescription, *Pneumocystis carinii* pneumonia prophylaxis, or *Mycobacterium avium* complex prophylaxis) at least 90 days apart.

## Discussion

In this analysis, nonadherence to any prescribed medication due to costs was associated with lack of recent and sustained HIV viral suppression. Addressing financial barriers to antiretroviral therapy (ART) adherence might improve levels of viral suppression, which is central to ending the HIV epidemic in this country (*6*). Cost saving–related nonadherence was not associated with race but was associated with having a household income above the poverty level. Persons with incomes above the poverty level might not be eligible for the Ryan White HIV/AIDS Program or other assistance programs that can reduce medication costs. Persons who were nonadherent due to prescription drug costs were more likely to seek care at emergency departments and be hospitalized, services that are more costly to the health care system than routine outpatient care. Further, persons who were nonadherent due to prescriptions drug costs were nearly twice as likely to not be engaged in HIV medical care, which might contribute to poorer health outcomes. Increasing the number of persons with HIV infection who are virally suppressed by reducing cost-related barriers to medication adherence might decrease morbidity, mortality, and risk for HIV transmission, as well as promote less costly health care utilization.

Many ART adherence interventions focus on changing patient behaviors, but reducing nonadherence due to costs might require increasing use of programs that provide affordable access to ART and reducing medication costs for the privately insured. Persons with private insurance, those without Medicaid, and those with an unmet need for ADAP were more likely to report nonadherence due to prescription drug costs. The U.S. Department of Health and Human Services has prioritized efforts to lower prescription drug prices and reduce out-of-pocket costs.^†^ Medicaid expansion has reduced the number of persons with HIV infection who are uninsured and is associated with an increase in the number of persons who are taking ART and are virally suppressed (*7*). CDC has provided information for state and local HIV prevention and care programs regarding Medicaid coverage for persons with HIV infection to improve access to care and medications (*8*). Case managers can assist persons with HIV infection obtain needed financial assistance for prescription medications. Cost-sharing assistance programs and patient assistance programs can help lower or eliminate the cost of ART for persons with HIV infection who are privately insured or who are not eligible for Medicaid or ADAP (*9*). The U.S. Department of Health and Human Services, in collaboration with pharmaceutical companies, the National Alliance of State and Territorial AIDS Directors, and community partners, developed a common enrollment tool to facilitate patient applications for patient assistance programs (*10*).

The prevalence of prescription nonadherence due to prescription drug costs among U.S. adults with HIV infection (7%) was similar to that in the U.S. adult population overall in 2013 (8%) (*3*). Fewer persons with HIV infection asked their doctors for a lower cost medication (9%) or used alternative therapies (2%) than did all U.S. adults (15% and 4%, respectively), and the prevalence of buying prescription drugs from another country was similar among persons with HIV infection (1%) and the population overall (2%). Nonadherence to prescribed medications can have negative consequences for all conditions. Because of the strong relationship between HIV infection and unsuppressed viral load, nonadherence among persons with HIV infection leads to increased morbidity, mortality, and risk for HIV transmission (*4*,*5*).

The findings in this report are subject to at least four limitations. First, self-reported information might be subject to biases that can result in measurement error. Recall and social desirability biases might underestimate adherence; therefore, these estimates of cost saving–related nonadherence should be viewed as minimum estimates. Second, the Medical Monitoring Project’s person-level response rate was low; however, the data were adjusted for nonresponse, which should reduce bias. Third, unmet need for ADAP was self-reported, and eligibility for ADAP was not assessed. Finally, interviewees were asked about nonadherence to all prescription drugs, not solely antiretroviral medications. However, among all persons taking prescription drugs in this study, 97% were taking ART, thus, these results are likely reflective of cost savings–related ART nonadherence. Nevertheless, adherence to all prescribed medications is important for optimal patient health outcomes.

Adults with HIV in the United States used various strategies to reduce prescriptions drug costs. The prevalence of nonadherence due to prescription drug costs among persons with HIV infection was similar to that among the overall U.S. population and was associated with poorer clinical outcomes, including reduced viral suppression rates and suboptimal medical care utilization. Removing barriers to ADAP and Medicaid coverage, in addition to reducing medication costs for persons with private insurance, could help to decrease nonadherence related to cost concerns, which will contribute to improved health outcomes among persons with HIV infection and decrease HIV transmission.

**Summary**

**What is already known about this topic?**

U.S. patients pay 14% of prescription drug costs out of pocket. Limited information exists about whether out-of-pocket costs for human immunodeficiency virus (HIV) medication are associated with treatment adherence.

**What is added by this report?**

Analysis of Medical Monitoring Project data found that approximately 14% of persons with HIV infection used prescription drug cost-saving strategies; 7% had cost saving–related nonadherence, which was associated with unmet need for the Ryan White AIDS Drug Assistance Program (ADAP), not having Medicaid, having private insurance, lower HIV medical care engagement, and lower viral suppression.

**What are the implications for public health practice?**

Removing barriers to ADAP and Medicaid and reducing private insurance medication costs might decrease cost saving–related nonadherence among persons with HIV infection and improve their health.

## Footnotes

* https://www.cdc.gov/hiv/statistics/systems/mmp/index.html.

† https://www.hhs.gov/sites/default/files/AmericanPatientsFirst.pdf.
